# Development and preliminary evaluation of Chinese Vitiligo Quality of Life Scale (CVQLS)

**DOI:** 10.3389/fpsyg.2025.1622757

**Published:** 2025-06-24

**Authors:** Bo Wang, Qi Wang, Zeqian Wang, Jia Zhang, Yuqi Zhou, Xiaoqi Chen, Tong Wu, Shishi Xiong, Xiaodong Jin, Shanshan Gao, Lei Shang, Chunying Li, Zhe Jian

**Affiliations:** ^1^Department of Dermatology, Xijing Hospital, Fourth Military Medical University, Xi’an, Shaanxi, China; ^2^Department of Health Statistics, School of Public Health, Fourth Military Medical University, Xi’an, Shaanxi, China

**Keywords:** vitiligo, quality of life, scale development, test–retest reliability, split-half reliability, construct validity, criterion-related validity, discriminative ability

## Abstract

**Introduction:**

Current research shows that there is no vitiligo quality-of-life measurement instrument suitable for Chinese patients. At present, the DLQI scale commonly used with vitiligo patients in China includes symptom dimensions or items that are not applicable to vitiligo patients. Therefore, it is necessary to develop a quality-of-life scale specific to vitiligo patients in China.

**Methods:**

In this study, the item pool was created through a comprehensive review of relevant literature, focus group discussions, and brainstorming. Two rounds of Delphi expert consultation and a semi-structured interview were conducted to modify the item pool and form the draft scale. Two rounds of questionnaire investigations were used to select items and form the final scale. The reliability, validity, and discriminative ability were evaluated based on the third round of questionnaire investigation.

**Results:**

The scale contains 3 dimensions and 25 items, and the total cumulative variance contribution rate was 64.54%. The Cronbach’s *α* coefficient was 0.972; the split-half reliability coefficient was 0.950, and the test–retest reliability coefficient was 0.776. The Spearman correlation coefficient with the Dermatology Life Quality Index (DLQI) was 0.650. The scores of the scale or each dimension were correlated with patient characteristics, including gender, disease course, disease stage, Body Surface Area (BSA), and white spot area.

**Conclusion:**

This study developed the Chinese Vitiligo Quality of Life Scale (CVQLS) to measure the quality of life of vitiligo patients in China. Compared to the commonly used DLQI, the CVQLS removed items related to skin disease symptoms while incorporating concerns specific to Chinese patients, such as the economic burden. The scale is thus tailored to the needs of Chinese vitiligo patients. Preliminary results indicate that the CVQLS has good reliability, validity, and discriminative ability.

## 1 Introduction

Vitiligo is a common acquired disorder of depigmentation characterized by amelanotic macules, with a prevalence of about 1% globally (Ezzedine et al., 2015). Although it is not life-threatening, it affects patients’ appearance and poses a chronic cosmetic issue. Vitiligo can cause patients to feel embarrassed, ashamed, and fearful of rejection (Mattoo et al., 2002). It carries significant stigma in some South Asian cultures. In India, vitiligo has been referred to as “Sweta Kustha,” which means “white leprosy” (Parsad et al., 2003). These negative impacts not only seriously decrease the quality of life for vitiligo patients, but can also lead to psychiatric problems such as depression or anxiety (Kent and Al’Abadie, 1996; Wakkee and Nijsten, 2009). Despite the negative views held by the general population, vitiligo, unlike many other skin conditions, typically presents with minimal or no skin irritation. Doctors cannot accurately estimate the degree of impact that vitiligo has on different patients based solely on symptoms, which can sometimes lead to communication conflicts between doctors and patients. Therefore, it is crucial to develop an instrument for accurately assessing the impact on the quality of life of vitiligo patients.

Currently, the assessment of quality of life for patients with vitiligo largely depends on general dermatological quality-of-life instruments, such as Skindex-16 (Chren et al., 2001) and Dermatology Life Quality Index (DLQI) (Finlay and Khan, 1994). However, these instruments include items assessing symptoms that are relatively less problematic in vitiligo patients, which may reduce their sensitivity. Several vitiligo quality-of-life scales have been developed, including the Vitiligo-specific-quality-of-life instrument (VitiQoL) (Lilly et al., 2013), Vitiligo Life Quality Index (VLQI) (Senol et al., 2013), Vitiligo Impact Scale-27 (VIS-27) (Ramam et al., 2013), and Vitiligo Impact Scale-22 (VIS-22) (Gupta et al., 2014). However, these scales were not designed for the Chinese population. Consequently, their items do not fully cover all quality-of-life concerns specific to Chinese vitiligo patients. Given China’s unique cultural and social context, these tools may not be appropriate for measuring the quality of life of vitiligo patients in China. Therefore, it is necessary to develop a vitiligo quality-of-life measurement instrument specifically tailored to the Chinese patients.

## 2 Methods

### 2.1 Participants

**Figure 1:**
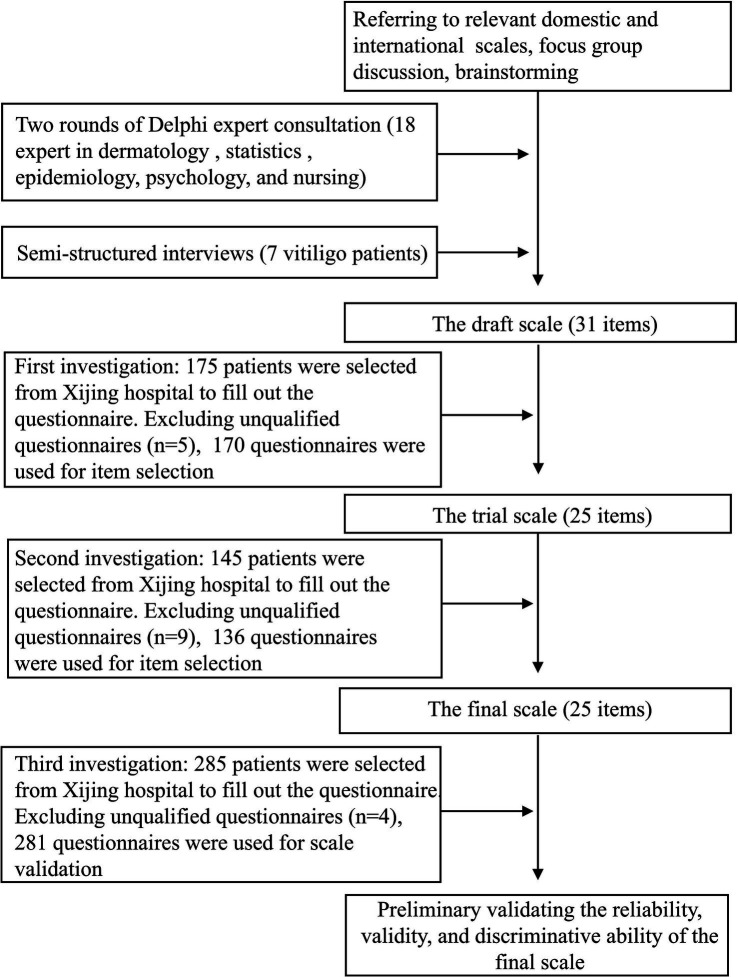
The flowchart of the research process for Chinese Vitiligo Quality of Life Scale (CVQLS).

The study was conducted from May to September 2024 in Shaanxi Province, China, with the aim of obtaining representative samples of individuals aged 18 to 65 years with vitiligo. An overview of the study procedure is shown in Figure 1.

The inclusion criteria for the subjects were as follows: participants had to be aged between 18 and 65 years, provide informed consent, have a clinical diagnosis of vitiligo, have no psychological disorders, and be able to complete the questionnaires. Participants were excluded from the study if they were illiterate or unwilling to participate.

The study utilized the Chinese Vitiligo Quality of Life Scale (CVQLS) and collected demographic information through a questionnaire that gathered data on age, gender, education level, marital status, and other relevant factors. Participants were invited to complete the questionnaire administered through mobile devices using QR code scanning in a designated room, under the guidance of a researcher. Prior to the survey, participants were provided with informed consent, and researchers clarified the purpose and significance of the study. All participants were assured that their information would remain confidential and completed informed consent forms.

### 2.2 Conceptual model and draft scale development

The conceptual model of the scale was developed after a comprehensive review of relevant domestic and international literature. The review focused particularly on the quality of life in individuals with vitiligo. After that, we gathered the item pool, by referencing established scales, including the Vitiligo-specific-quality-of-life instrument (VitiQoL), Dermatology Life Quality Index (DLQI), Skindex-16, Vitiligo Impact Scale-22 (VIS-22), Rosa Quality of Life (RosaQoL) (Tannus et al., 2018), Vitiligo Patient Satisfaction (VIPS) (Salzes et al., 2016), and Vitiligo Life Quality Index (VLQI). We had a focus group discussion to ensure that the item pool adequately encompassed the connotations and scope of the conceptual model. Subsequently, the Delphi consultation method was used to assess the validity of the gathered information. Experts from various fields, including dermatology (11), statistics (1), epidemiology (1), psychology (3), and nursing (2), participated in two rounds of the Delphi consultation. We then conducted focus group discussion again to modify the items’ contents. The item pool was then assessed through semi-structured interviews with 7 participants diagnosed with vitiligo, who were selected from the outpatient departments of Xijing Hospital, to ensure that all aspects of the patients’ impaired quality of life were covered by the item pool without duplication. Finally, a draft scale with 31 items was formed after the third focus group discussion.

### 2.3 Scoring methods

Each item of the scale assessed the impact of vitiligo on quality of life over the preceding month. Five option levels were established for various items through dimensional analysis. Namely: not at all, a little, moderately, very, and extremely. These options were assigned numerical values from 0 to 4, respectively. Total scores are calculated by summing the scores of all items. A higher score indicates a poorer quality of life.

### 2.4 Investigation methods

First, the researcher explained the purpose and procedure of the study and the significance of the questionnaire to the patients who agreed to participate. Participants were asked to complete a questionnaire via a QR code about the impact of vitiligo on their quality of life in the past month. Finally, the researcher collected the data from the completed questionnaires and then analyzed the data.

### 2.5 Constructing the trial scale (first investigation)

Sample 1 consisted of 175 patients sampled from the Department of Dermatology of Xijing Hospital, using predetermined survey methods and inclusion/exclusion criteria. After all questionnaires were completed, the researcher analyzed the results. This sample was used to refine items from the draft scale and to construct the trial scale.

### 2.6 Constructing the final scale (second investigation)

Sample 2 consisted of 145 patients sampled from the Department of Dermatology of Xijing Hospital, using the same method and inclusion/exclusion criteria as Sample 1. After all questionnaires were completed, the researcher analyzed the results. This sample was used to refine items from the trial scale and to construct the final scale.

### 2.7 Evaluating the final scale (third investigation)

Sample 3 consisted of 285 patients sampled from the Department of Dermatology of Xijing Hospital, using the same method and inclusion/exclusion criteria as Sample 1. After all participants completed the questionnaires, the researcher analyzed the results to assess the scale’s reliability, validity, and discriminative ability. Additionally, to assess the test–retest reliability, 43 patients were selected to complete the questionnaire a second time 2 weeks later.

### 2.8 Quality control methods

All questionnaires filled out by participants were web-based and appeared after scanning the QR code. To avoid invalid questionnaires due to participant omissions, the web-based questionnaire was set to not allow submission if any questions were left unanswered. Then, the questionnaire data were exported into an Excel spreadsheet. If a questionnaire had identical responses for all items, it was excluded from the sample. The researchers carefully checked the data and eliminated anyone unqualified. The questionnaire data were imported into SPSS 26.0 for analysis.

### 2.9 Statistical methods

#### 2.9.1 Item selection

The following 5 methods were used for item selection (Xu et al., 2023). (1) The critical ratio analysis method: After computing and ordering the total scores from the scale, the critical scores separating the upper and lower 27% of respondents were identified. The scales were divided into two distinct groups based on these critical scores. An independent sample *t*-test was conducted to compare the high-score group with the low-score group, discarding items with a *p*-value > 0.05. (2) The discrete trend method: Items with a standard deviation <0.85 of the score were discarded. (3) The correlation coefficient method: Items showing a correlation coefficient <0.4 with the total score were discarded. (4) The exploratory factor analysis (EFA) method: Items with factor loading values <0.4 or with factor loading values > 0.4 on more than one factor were discarded. (5) Cronbach’s *α* coefficient method: After calculating the Cronbach’s *α* coefficient for all items, those that reduced the overall alpha level were discarded. Based on these methods, the following item exclusion principles were applied: An item was deleted if discarded by two or more methods. If an item was discarded by one method, it was subject to deletion or modification based on focus group discussion and expert opinions.

#### 2.9.2 Reliability analysis

Reliability was evaluated with Cronbach’s *α* coefficient, test–retest reliability coefficient, and split-half reliability coefficient. Coefficient values for the total scale and dimensions equal to or greater than 0.70 were considered satisfactory (Zakariya, 2022).

#### 2.9.3 Validity analysis

Validity was evaluated with convergent validity, discriminant validity, criterion-related validity, and construct validity. Convergent validity was evaluated using the average variance extracted (*AVE*). All dimensions had *AVE* values greater than 0.5, suggesting satisfactory convergent validity (Salameh, 2022). Discriminant validity was ascertained with the finding that the square root of *AVE* was larger than the correlation between each pair of constructs (Zhang et al., 2019). Criterion-related validity was assessed by calculating the Spearman correlation coefficient between the total score of our scale and the DLQI. The correlation coefficient greater than 0.7 was considered strong. Confirmatory factor analysis was conducted to validate the results from the EFA. To assess the model fit, the following absolute and incremental fit indices were used: *χ*^2^/*df*, the root mean square error of approximation (*RMSEA*), the goodness-of-fit index (*GFI*), the comparative fit index (*CFI*), the normed fit index (*NFI*), and the non-normed fit index (*NNFI*). The *GFI*, *CFI*, *NFI*, and *NNFI* values > 0.90 indicated a good fit. Values from 0.8 to 0.9 were considered acceptable. An *RMSEA* value < 0.05 suggested a good fit, with values from 0.05 to 0.08 being acceptable. A *χ*^2^/*df* value < 3 indicated a good fit, with values from 3 to 5 being acceptable (Beran and Violato, 2010; Su et al., 2015).

#### 2.9.4 Discriminative ability analysis

Discriminative ability evaluated significant differences in scores of the total scale and dimensions with different characteristics. Two-sample *t*-tests were conducted to examine differences in scores based on individual characteristics such as gender, nationality, disease activity, and the area of white spots. While one-way ANOVA was conducted to examine differences in scores based on individual characteristics such as age, illness duration, education level, marital status, disease classification, and BSA. Where appropriate, post-hoc tests were conducted using Fisher’s LSD-*t* test. The Levene statistic was used to test the homogeneity of group variances. When equal variances were not assumed, corrections were applied using the Welch test and Games Howell *post hoc* test (*p* < 0.05). Quantitative data were expressed as the mean ± standard deviation (*SD*), and qualitative data were expressed as numbers and percentages. All statistical analyses were performed using SPSS 26.0. The general characteristics of participants are shown in Table 1.

**Table 1:** General characteristics of vitiligo patients in samples collected from three rounds of investigation.

Group	Sample 1 (*n* = 170)		Sample 2 (*n* = 136)		Sample 3 (*n* = 281)	
	*n*	%	*n*	%	*n*	%
Gender						
Male	77	45.29	63	46.32	157	55.87
Female	93	54.71	73	53.68	124	44.13
Age group (years old)						
18–29	67	39.41	48	35.3	88	31.32
30–39	55	32.35	42	30.88	90	32.03
40–49	19	11.18	17	12.5	46	16.37
> = 50	29	17.06	29	21.32	57	20.28
Ethnic group						
The Han ethnic group	170	100	135	99.26	274	97.51
Other ethnic groups	0	0	1	0.74	7	2.49
Marital status						
Unmarried	76	44.71	50	36.76	79	28.11
Married	94	55.29	85	62.5	199	70.82
Divorced	0	0	1	0.74	3	1.07
Widowed	0	0	0	0	0	0
Disease course (months)						
<12	50	29.41	37	27.2	73	25.98
12–59	49	28.82	36	26.47	121	43.06
60–119	30	17.65	22	16.18	33	11.74
> = 120	41	24.12	41	30.15	54	19.22
Type						
Segmental	32	18.82	15	11.03	27	9.61
Nonsegmental	133	78.24	118	86.76	251	89.32
Mixed	4	2.35	2	1.47	3	1.07
Unclassified	1	0.59	1	0.74	0	0
Stage						
Active	140	82.35	115	84.56	210	74.73
Stable	30	17.65	21	15.44	71	25.27
BSA						
<1	81	47.65	54	39.7	148	52.67
1–5	72	42.35	43	31.62	117	41.64
>5 and <=50	16	9.41	22	16.18	16	5.69
>50	1	0.59	17	12.5	0	0
White spot area						
Exposed areas (Face, neck, hands)	140	82.35	110	80.88	230	81.85
Non exposed areas	30	17.65	26	19.12	51	18.15

## 3 Results

### 3.1 General characteristics of participants

For Sample 1, 175 questionnaires were distributed via convenience sampling, all of which were returned (response rate 100%), with 170 valid responses (validity rate 97.1%). For Sample 2, all 145 distributed questionnaires were returned (response rate 100%), yielding 136 valid responses (validity rate 93.8%). For Sample 3, 285 questionnaires were distributed and fully returned (response rate 100%), of which 281 were valid (validity rate 98.6%).

### 3.2 Item selection

Data from Sample 1 were used for item selection. According to the exclusion criteria, 6 items were deleted, leaving 25 items for the trial scale. Data from Sample 2 were used for item selection as well. Using the same exclusion criteria, no items were deleted in Sample 2, confirming all 25 items as the final scale.

### 3.3 Structure of the scale

EFA was used to determine the potential factor structure of the items utilized in Sample 2. The Kaiser-Meyer-Olkin (KMO) measure for the sample was 0.930 (greater than 0.6), and the approximate chi-squared value obtained from Bartlett’s test of Sphericity was 2,404.83 (*p* < 0.05), confirming the suitability of the data for EFA. EFA was conducted using principal component analysis and maximum variance. All items had loadings of 0.40 or higher, and all factors had eigenvalues of 1 or greater, indicating that these factors and items met the required contribution level to the scale. The three factors identified through EFA were taken as the preliminary dimensions of the scale (Table 2). Factor 1, explaining 52.133% of the variance, was assigned as Daily Life Restriction; Factor 2, explaining 6.427%, as Disease Burden; and Factor 3, explaining 5.984%, as Social Limitation. Together, these three factors accounted for 64.544% of the variance in the 24 items (excluding the 25th self-evaluation item).

**Table 2:** Item loadings of the final scale.

Item	Daily Life Restriction	Disease Burden	Social Limitation
Q1	0.708		
Q2			0.672
Q3			0.735
Q4			0.728
Q5			0.772
Q6			0.724
Q7		0.628	
Q8		0.752	
Q9		0.642	
Q10		0.709	
Q11		0.63	
Q12	0.638		
Q13	0.742		
Q14	0.667		
Q15	0.681		
Q16	0.691		
Q17	0.669		
Q18	0.631		
Q19	0.719		
Q20	0.635		
Q21	0.296		
Q22		0.709	
Q23		0.724	
Q24		0.695	
Eigenvalue	12.512	1.543	1.436
Variance Contribution Rate (%)	52.133	6.427	5.984

The item loadings that did not reach the threshold (>0.4) have been omitted from the table.

### 3.4 Reliability

Reliability was established using the data from Sample 3. The Cronbach’s *α* coefficient for the total scale was 0.972, with the 3 factors ranging from 0.924 to 0.954. The split-half reliability of the total scale was 0.950, with the 3 factors ranging from 0.894 to 0.921. The two-week test–retest reliability for the total scale (*n* = 43) was 0.776, with the 3 factors ranging from 0.711 to 0.823 (Table 3). These results indicate that the scale has satisfactory reliability.

**Table 3:** Reliability coefficients of the final scale.

Dimension	Total	Daily Life Restriction	Disease Burden	Social Limitation
Cronbach’s α coefficient	0.972	0.954	0.945	0.924
Split-half reliability coefficient	0.950	0.921	0.894	0.901
Test–retest reliability coefficient	0.776	0.743	0.711	0.823

### 3.5 Validity

On average, participants of Sample 3 (*n* = 281) spent 3.92 ± 1.92 min completing the scale. The Spearman correlation coefficient with the DLQI as the validity criterion was 0.650. A validity coefficient larger than 0.60 indicates acceptable criterion-related validity.

The confirmatory factor analysis of Sample 3 was used to evaluate the factor structure of the scale. The results (*χ*^2^/*df* = 2.716 < 3, *RMSEA* = 0.078, *GFI* = 0.820, *CFI* = 0.931 > 0.9, *NFI* = 0.895, *NNFI* = 0.923 > 0.9) suggested a good fit model, except for *RMSEA*, *GFI*, and *NFI*, which did not meet the optimal criteria but were within acceptable ranges (*RMSEA* = 0.078 < 0.08, *GFI* = 0.820 > 0.8, *NFI* = 0.895 > 0.8). These results show that the scale has good construct validity.

As shown in Table 4, the factor loading of each item was above 0.70 in the model. *AVE* was above 0.50, and *CR* was above 0.80 for all 3 factors. These results supported good convergent validity of the scale. Table 5 shows the square root of *AVE* for the 3 factors. According to the results in Table 5, the square root of *AVE* was greater than the correlation coefficients between the factors, indicating good discriminant validity.

**Table 4:** Convergence validity of the final scale.

Item		Dimension	Estimate	*AVE*	*CR*
Q1	<−--	Daily Life Restriction	0.834	0.676	0.954
Q12	<−--		0.873		
Q13	<−--		0.816		
Q14	<−--		0.769		
Q15	<−--		0.819		
Q16	<−--		0.852		
Q17	<−--		0.815		
Q18	<−--		0.824		
Q19	<−--		0.799		
Q20	<−--		0.817		
Q7	<−--	Disease Burden	0.868	0.660	0.946
Q8	<−--		0.836		
Q9	<−--		0.755		
Q10	<−--		0.875		
Q11	<−--		0.833		
Q21	<−--		0.838		
Q22	<−--		0.800		
Q23	<−--		0.783		
Q24	<−--		0.706		
Q2	<−--	Social Limitation	0.824	0.707	0.924
Q3	<−--		0.818		
Q4	<−--		0.872		
Q5	<−--		0.846		
Q6	<−--		0.844		

**Table 5:** Discriminant validity of the final scale.

Dimension	Daily Life Restriction	Disease Burden	Social Limitation
Daily Life Restriction	0.822	–	–
Disease Burden	0.809	0.812	–
Social Limitation	0.764	0.811	0.840

The numbers on the diagonal were the square root of the average variance extraction (AVE).

### 3.6 Discriminative ability

The discriminative ability of Sample 3 (*n* = 281) is shown in Table 6. Significant differences in the Disease Burden dimension scores were observed for patients of different disease course groups (*p* < 0.05). Similarly, significant differences were found in the total scores and Daily Life Restriction/Disease Burden dimension scores for patients of different gender groups (*p* < 0.05). Significant differences were found in the total scores and Daily Life Restriction/Social Limitation dimension scores for patients of different disease stage groups (*p* < 0.05). Additionally, significant differences were noted in the total scores and Disease Burden/Social Limitation dimension scores for patients of different BSA groups (*p* < 0.05). Furthermore, significant differences were observed in the total scores and Disease Burden dimension scores for patients of different White spot area groups (*p* < 0.05). While no significant differences were observed in the total scores or dimension scores for patients of different age, ethnic, marital status, and disease type groups (*p* > 0.05). These results indicate that the scale has good discriminative ability.

**Table 6:** Comparison of each dimension score of final scale reported by different characteristics of patients in the third questionnaire investigation (*n* = 281).

Group	Total	Daily Life Restriction	Disease Burden	Social Limitation
Gender				
Male	40.06 ± 26.96	12.94 ± 11.13	17.66 ± 11.25	7.60 ± 6.02
Female	47.90 ± 25.30^a^	15.85 ± 11.36^a^	21.57 ± 9.79^a^	8.40 ± 5.65
*p*-value	0.013	0.032	0.002	0.259
Age (years old)				
18–29	44.11 ± 26.79	15.27 ± 11.81	19.01 ± 10.00	7.90 ± 5.99
30–39	44.02 ± 26.12	14.29 ± 10.83	19.80 ± 11.34	8.12 ± 5.62
40–49	41.07 ± 23.29	12.57 ± 9.52	18.63 ± 9.56	7.76 ± 5.63
> = 50	43.79 ± 29.41	13.82 ± 12.63	19.91 ± 12.15	7.91 ± 6.37
*p*-value	0.925	0.613	0.893	0.988
Ethnic				
The Han ethnic group	43.26 ± 26.68	14.11 ± 11.36	19.24 ± 10.82	7.93 ± 5.91
Other ethnic groups	53.57 ± 14.64	18.43 ± 8.64	24.86 ± 8.40	8.57 ± 4.24
*p*-value	0.310	0.320	0.175	0.777
Marital status				
Unmarried	44.25 ± 27.31	15.30 ± 12.13	18.84 ± 10.31	8.15 ± 5.84
Married	43.56 ± 26.16	13.93 ± 10.98	19.74 ± 10.92	7.92 ± 5.91
Divorced	21.67 ± 23.76	5.00 ± 6.08	10.00 ± 13.89	4.67 ± 3.06
Widowed	–	–	–	–
*p*-value	0.350	0.241	0.261	0.597
Disease course (months)				
<12	42.38 ± 28.27	14.59 ± 11.95	18.56 ± 11.30	7.38 ± 5.91
12–59	40.09 ± 28.50	13.15 ± 12.20	17.68 ± 11.35	7.35 ± 6.18
60–119	48.06 ± 18.98	14.91 ± 8.11	21.76 ± 8.03	9.36 ± 5.57
> = 120	49.96 ± 21.85	15.70 ± 9.98	22.87 ± 9.34^b^	9.20 ± 5.03
*p-*value	0.057	0.512	0.007	0.096
Disease type				
Segmental	47.33 ± 28.10	14.70 ± 11.91	21.22 ± 11.20	9.33 ± 6.78
Nonsegmental	43.34 ± 26.39	14.25 ± 11.29	19.27 ± 10.78	7.85 ± 5.77
Mixed	24.33 ± 9.29	7.00 ± 7.00	12.33 ± 5.51	3.67 ± 3.06
Unclassified	–	–	–	–
*p-*value	0.343	0.530	0.352	0.205
Disease stage				
Active	45.89 ± 26.92	15.35 ± 11.64	20.10 ± 10.69	8.43 ± 5.92
Stable	36.51 ± 23.96^c^	10.89 ± 9.58^c^	17.28 ± 10.88	6.52 ± 5.51^c^
*p-*value	0.010	0.002	0.057	0.017
BSA				
<1	39.36 ± 26.57	12.97 ± 11.32	17.59 ± 10.65	7.07 ± 5.88
1–5	47.76 ± 26.00^d^	15.52 ± 11.34	21.23 ± 10.75^d^	8.83 ± 5.72^d^
>5 and <=50	50.94 ± 23.96	16.31 ± 10.20	22.44 ± 9.96	9.63 ± 5.81
>50	–	–	–	–
*p-*value	0.019	0.141	0.012	0.026
White spot area				
Exposed areas (Face, neck, hands)	45.14 ± 26.51	14.84 ± 11.36	20.04 ± 10.71	8.25 ± 5.89
Non exposed areas	36.20 ± 25.33^e^	11.41 ± 10.73	16.43 ± 10.73^e^	6.59 ± 5.63
*p-*value	0.029	0.050	0.030	0.067

a: *p* < 0.05 vs. Male. b: *p* < 0.05 vs. 12–59. c: *p* < 0.05 vs. Active. d: *p* < 0.05 vs. <1. e: *p* < 0.05 vs. Exposed areas (Face, neck, hands).

## 4 Discussion

The development of this scale adheres strictly to classical measurement theory. Our study established an item pool by a comprehensive review of relevant literature, focus group discussions and brainstorming. Two rounds of Delphi expert consultation and semi-structured interviews with vitiligo patients were conducted to modify the item pool and form a draft scale. Two rounds of questionnaire investigations were used to select items and form the final scale. Based on the third round of investigation, the reliability, validity, and discriminative ability of the scale were evaluated and satisfactory results were obtained. The evaluation results indicate that the scale complies with the standards of metrology.

Current research indicates that DLQI is the predominant instrument for assessing the quality of life among vitiligo patients in China, and its Chinese version is widely acknowledged by Chinese scholars. DLQI has 6 dimensions: Symptoms/Feelings, Daily Activities, Leisure, Work/School, Personal Relationships and Treatment. However, the skin symptoms of vitiligo patients are usually not prominent, making the use of the Symptoms/Feelings dimension unsuitable for assessing vitiligo. The 3 dimensions of CVQLS (Daily Life Restriction, Disease Burden and Social Limitation) represent the aspects that have the greatest impact on the quality of life of vitiligo patients. Besides, it added items that were not covered by DLQI such as the economic burden (e.g., Q22), concern about disease refractory (e.g., Q24), and pressure from recurrence (e.g., Q23). Therefore, CVQLS is more suitable for measuring the quality of life of vitiligo patients in Chinese population than DLQI.

In recent years, international scholars have developed several vitiligo quality-of-life scales, such as VitiQoL, VLQI, VIS-27, and VIS-22. Among these, VitiQoL is a more commonly used scale. However, it was developed based on a U.S. population and, due to cultural differences, its item composition does not fully cover aspects that are of particular concern to Chinese vitiligo patients, such as the impact on family, work, and learning, as well as the burden of time and financial costs, and the pressure of disease recurrence and incurability. Therefore, it is not directly applicable to the Chinese population. Moreover, the development process of VitiQoL is incomplete, involving only a single round of questionnaire surveys with a total of 90 patients. It lacks important steps such as item selection, confirmatory factor analysis, and test–retest reliability evaluation, which are essential for meeting measurement requirements. Similar cultural differences and developmental shortcomings are observed in several other foreign vitiligo quality-of-life scales. In contrast, the development of the Chinese Vitiligo Quality of Life Scale (CVQLS) strictly adheres to the standard scale development process, ensuring a more comprehensive approach. The CVQLS addresses key concerns of Chinese vitiligo patients that are not covered by VitiQoL, such as family impact (e.g., Q9), occupational and learning impact (e.g., Q16), time and financial burden (e.g., Q22), and the pressure of disease recurrence and refractory (e.g., Q23 and Q24). In summary, the CVQLS is more scientifically rigorous and better suited for the Chinese population.

Our study found that female patients scored higher than male patients, indicating that vitiligo had a greater impact on the quality of life of female patients than male patients. These findings were similar to those of other studies (Ongenae et al., 2005b; Linthorst Homan et al., 2009), which suggested that female vitiligo patients had a poorer quality of life compared to males. The scores of patients with active vitiligo were higher than those of patients with stable vitiligo, indicating a poorer quality of life for patients with active conditions. Studies by Silpa-Archa et al. (2020) and Karelson et al. (2013) also demonstrated that active vitiligo patients had a greater impact on their quality of life than those with stable vitiligo. The larger the BSA, the higher the score of vitiligo patients, indicating a poorer quality of life with larger vitiligo areas. These results were similar to those of Radtke et al. (2009) and Bae et al. (2018), which suggested significantly higher reductions in quality of life with larger affected areas. Patients with vitiligo on exposed areas, such as the face, neck, and hands, scored higher, indicating that vitiligo on exposed areas had a greater impact on quality of life. A study by Ongenae et al. (2005a) also showed similar results, suggesting that vitiligo on exposed areas, especially the face, caused greater damage to quality of life. Karelson et al. (2013) also found that patients with vitiligo on the hands had a higher impact on their quality of life.

The participants in this study are all patients from Xijing Hospital in Xi’an, Shaanxi Province, a tertiary Grade A hospital with abundant medical resources. The dermatology department of the hospital is affiliated with the Vitiligo Research Center of the Chinese Medical Association and operates a specialized clinic for vitiligo, attracting patients from all over China. Participants in this study come from 69 cities and 20 provinces, municipalities, and autonomous regions in China. Therefore, we believe the participants in this study are representative of the general Chinese population. Based on this, we anticipate that the application scope of this tool can be expanded to various regions across China in the future.

This study has several limitations. Firstly, the participants were all patients who voluntarily sought medical treatment at the hospital, potentially excluding those less inclined to seek healthcare. This limitation is difficult to avoid in the practical questionnaire survey process. Next, to ensure the accuracy and reliability of the research results, we excluded illiterate patients from our sample. Although we did not encounter any illiterate patients during the questionnaire survey, this decision indeed reduces the universality of the scale. We recommend that users exercise caution when applying the scale to low-literacy populations. In future research, it may be worth considering the introduction of interpreters or the development of standardized audio versions to address this limitation. Then, all questionnaires were completed by participants on their mobile phones under the guidance of researchers. Compared to manual completion, differences in participants’ proficiency in using mobile phones may affect the results. Finally, the included sample size has just reached the required standard, and the majority of the sample consists of Han Chinese participants. Given the cultural differences between different ethnic groups and considering that a larger sample size may enhance credibility, future research should include larger and more diverse samples to further validate the practicality of the scale.

## 5 Conclusion

This study developed a measurement instrument named the Chinese Vitiligo Quality of Life Scale (CVQLS) in Table 7 (The English version) and Supplementary Table S1 (The Chinese version), which consists of 3 dimensions—Daily Life Restriction, Disease Burden and Social Limitation—and 25 items. Compared to the commonly used DLQI, CVQLS removed items related to skin disease symptoms while incorporating vitiligo-specific concerns in China, such as the economic burden, concern about disease refractory, and pressure from recurrence. It is therefore more suitable for Chinese vitiligo patients. Future research should test and confirm the CVQLS in diverse Chinese populations with larger sample sizes. Most importantly, the clinical application value of the CVQLS needs to be further explored and developed through future research.

**Table 7:** The CVQLS instrument.

CVQLS					
This scale is used to measure the impact of vitiligo on your quality of life in the past month.	Not at all → Extremely (0 → 4)				
	0	1	2	3	4
1. Vitiligo affects my daily activities.	□	□	□	□	□
2. Vitiligo affects my participation in social activities.	□	□	□	□	□
3. Vitiligo makes it difficult for me to be intimate with people.	□	□	□	□	□
4. Others may discriminate against with me because of my vitiligo.	□	□	□	□	□
5. Others may worry that my vitiligo is an infectious disease.	□	□	□	□	□
6. Others around me may feel uncomfortable because of my vitiligo.	□	□	□	□	□
7. I sometimes feel frustrated because of my vitiligo.	□	□	□	□	□
8. I always keep thinking about my vitiligo, making it difficult to concentrate on anything else.	□	□	□	□	□
9. I am worrying that my vitiligo may be passed on to children.	□	□	□	□	□
10. I hate living with vitiligo.	□	□	□	□	□
11. I do not want others to know my vitiligo.	□	□	□	□	□
12. Vitiligo has affected my clothing.	□	□	□	□	□
13. Vitiligo has affected my daily grooming practices (i.e., hairstyle, use of cosmetics).	□	□	□	□	□
14. Vitiligo has affected my daily sun protection measures during recreation (i.e., limiting exposure time during peak sun hours, seeking shade, wearing hat, long sleeves or pants).	□	□	□	□	□
15. Vitiligo makes me afraid to look in the mirror.	□	□	□	□	□
16. Vitiligo sometimes affects my ability to complete daily works or studies.	□	□	□	□	□
17. Vitiligo sometimes causes me insomnia or nightmares.	□	□	□	□	□
18. Vitiligo makes me afraid of intense sunshine.	□	□	□	□	□
19. Vitiligo makes me unwilling to participate in sports activities.	□	□	□	□	□
20. Vitiligo has affected my overall physical health.	□	□	□	□	□
21. I am worrying about the progression or spread of my vitiligo to new areas of the body.	□	□	□	□	□
22. The amounts of time and money spent on vitiligo treatment bother me a lot.	□	□	□	□	□
23. The recurrence of vitiligo bothers me a lot.	□	□	□	□	□
24. I think vitiligo is an incurable disease.	□	□	□	□	□
Please check how severe you currently feel your vitiligo is:					
25. Severity of my vitiligo.	□	□	□	□	□

## Data Availability

The data analyzed in this study is subject to the following licenses/restrictions: the data used in this study involves patient privacy and cannot be disclosed due to ethical and legal reasons. If readers want to obtain relevant data for this study, please contact the corresponding author. Requests to access these datasets should be directed to Zhe Jian, Email: xjzhejian@fmmu.edu.cn.
