# Supplementary material for: Development and preliminary evaluation of Chinese Vitiligo Quality of Life Scale (CVQLS)
Source: Front Psychol. 2025 Jun 24;16:1622757. doi: 10.3389/fpsyg.2025.1622757 (PMC12236179; doi:10.3389/fpsyg.2025.1622757)
Supplement: Supplementary file 1 [file Table_1.docx]

TABLE S1. The Chinese version of CVQLS instrument.

| CVQLS | | | | | |  |
| --- | --- | --- | --- | --- | --- | --- |
| 本量表用于评价近一个月白癜风对您生活质量的影响，请仔细阅读每一个问题，并在相应的方框中画“√” | | 完全没有→非常严重（0→4） | | | | |
|  | 0 | 1 | 2 | 3 | 4 |  |
| 1.白癜风影响了我的日常活动 | □ | □ | □ | □ | □ |  |
| 2.白癜风影响我参加社交活动 | □ | □ | □ | □ | □ |  |
| 3.白癜风使我难以与人亲昵 | □ | □ | □ | □ | □ |  |
| 4.我有时会担心他人因为白癜风歧视我 | □ | □ | □ | □ | □ |  |
| 5.其他人会担心我的白癜风是一种传染病 | □ | □ | □ | □ | □ |  |
| 6.我的白癜风会使周围的人感到不适 | □ | □ | □ | □ | □ |  |
| 7.白癜风有时会使我感到沮丧 | □ | □ | □ | □ | □ |  |
| 8.我总是在想我的白癜风使我难以集中注意力做事情 | □ | □ | □ | □ | □ |  |
| 9.我担心白癜风会遗传给孩子 | □ | □ | □ | □ | □ |  |
| 10.我讨厌带病（白癜风）生活 | □ | □ | □ | □ | □ |  |
| 11.我不想别人知道我患有白癜风 | □ | □ | □ | □ | □ |  |
| 12.白癜风影响了我的穿着 | □ | □ | □ | □ | □ |  |
| 13.白癜风影响了我的日常妆容（如发型、化妆品的使用） | □ | □ | □ | □ | □ |  |
| 14.白癜风影响了我的日常防晒措施（比如减少皮肤在强烈阳光下的暴露时间、寻找阴凉处、戴帽子、穿长袖或长裤等） | □ | □ | □ | □ | □ |  |
| 15.白癜风使我害怕照镜子 | □ | □ | □ | □ | □ |  |
| 16.白癜风有时会影响我完成某些日常的工作或学习 | □ | □ | □ | □ | □ |  |
| 17.白癜风有时会使我失眠或做噩梦 | □ | □ | □ | □ | □ |  |
| 18.白癜风使我害怕阳光明媚的好天气 | □ | □ | □ | □ | □ |  |
| 19.白癜风使我不愿意参加体育运动 | □ | □ | □ | □ | □ |  |
| 20.因为白癜风我感觉整个身体的健康状况都变差了 | □ | □ | □ | □ | □ |  |
| 21.我时刻在担心白癜风进展或者扩散到身体的其他新区域 | □ | □ | □ | □ | □ |  |
| 22.白癜风治疗花费的时间和金钱使我很困扰 | □ | □ | □ | □ | □ |  |
| 23.白癜风的复发使我很困扰 | □ | □ | □ | □ | □ |  |
| 24.我认为白癜风是无法治愈的疾病 | □ | □ | □ | □ | □ |  |
| **请检查一下目前自身的白癜风状况** |  |  |  |  |  |  |
| 25.我的白癜风严重程度 | □ | □ | □ | □ | □ |  |
